# True Digital Artery Aneurysm: A Case Report

**DOI:** 10.3400/avd.cr.25-00096

**Published:** 2025-11-15

**Authors:** Hiroki Nakabori, Hideyasu Ueda, Kenji Iino

**Affiliations:** Department of Cardiovascular Surgery, Kanazawa University, Kanazawa, Ishikawa, Japan

**Keywords:** digital artery aneurysm, true aneurysm, hand surgery

## Abstract

True aneurysms of the digital artery are extremely rare, and only several dozen cases have been reported worldwide. A 29-year-old man presented with a pulsatile, tender nodule in his left index finger. Angiography revealed a 7-mm saccular aneurysm of the proper palmar digital artery with well-developed distal collaterals. Under local anesthesia, the aneurysm was excised following proximal and distal ligation. It was histopathologically confirmed as a true aneurysm. Postoperatively, symptoms resolved without ischemic or neurological complications and without recurrence after 1 year. Thus, simple ligation and excision are effective when collateral circulation is sufficient.

## Introduction

Peripheral arterial aneurysms are rare and even rarer in the upper extremity than in the lower extremity, accounting for <1% of all peripheral aneurysms.^1)^ Among these aneurysms, digital artery aneurysms are exceedingly rare, with only several dozen cases reported globally. Here, we present a case of a true digital artery aneurysm in a young adult.

## Case Report

**Fig. 1:**
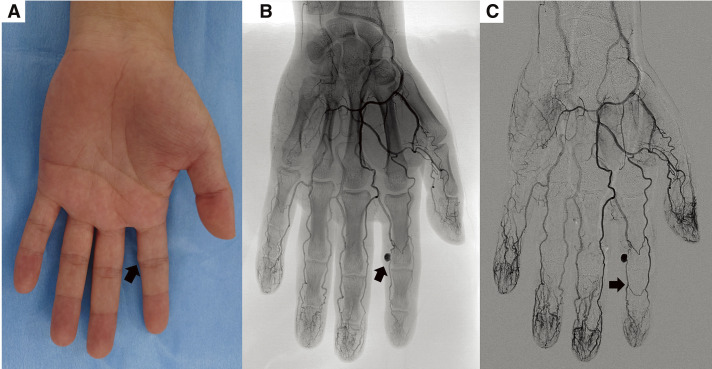
Preoperative photograph and angiography of the left hand. (**A**) A 7-mm pulsatile mass (arrow) on the ulnar side of the left index finger at the proximal interphalangeal joint. The lesion was elastic, mobile, and mildly tender. (**B**) Angiogram showing a 7-mm saccular aneurysm of the proper palmar digital artery at the distal portion of the proximal phalanx (arrow), without arteriovenous fistula. (**C**) Digital subtraction angiogram revealing a well-developed collateral circulation distal to the aneurysm (arrow).

**Fig. 2:**
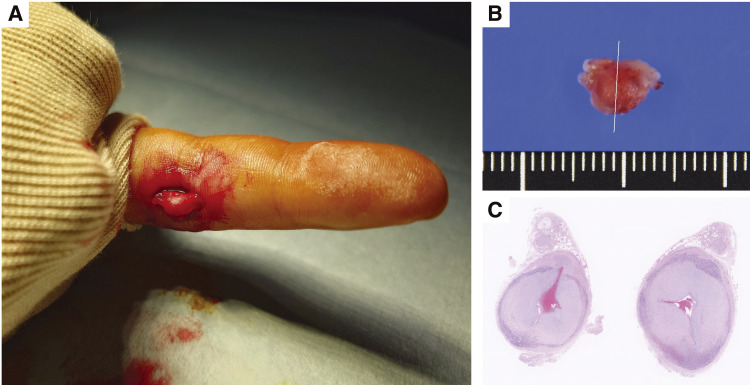
Intraoperative and pathological photographs. (**A**) Intraoperative view showing dissection of the aneurysm with isolation of the inflow and outflow arteries. (**B**) Resected specimen measuring 8 × 6 × 4 mm. (**C**) Histopathological examination showing marked intimal thickening, consistent with a true aneurysm.

A 29-year-old man presented with discomfort and tenderness in his left index finger. Approximately 1 month earlier, he had noticed a mildly tender, pulsatile nodule at the proximal interphalangeal joint and consulted a local physician (**Fig. 1A**). Ultrasonography revealed a structure continuous with the artery, suggestive of an aneurysm, and he was referred to our department. He worked as a freight train operator and was right-handed, with no history of trauma, comorbidities, surgery, or medication use. He denied recurrent irritation of the affected finger. Angiography revealed a 7-mm saccular aneurysm in the proper palmar digital artery at the distal end of the proximal phalanx of his left index finger. No arteriovenous fistula was present, and the distal collateral circulation was well developed (**Figs. 1B** and **1C**). He was diagnosed with a true digital artery aneurysm and opted for surgery. Under local anesthesia, a 3-cm incision was made over the aneurysm, which was then exposed (**Fig. 2A**). Before transection, the inflow and outflow arteries were isolated and doubly ligated with 3-0 silk sutures, and the aneurysm was then resected (**Fig. 2B**).

Histopathological examination revealed marked intimal thickening with disruption of the internal and external elastic laminae of the aneurysmal wall. The adventitia showed histiocytic and lymphocytic infiltration with neovascularization, consistent with reactive change. No malignant features were observed, confirming a true aneurysm (**Fig. 2C**). Discomfort and tenderness resolved completely after surgery. No ischemic or neurological complications occurred, and the patient remained recurrence-free at 1 year.

## Discussion

Peripheral arterial aneurysms are very rare, with upper extremity lesions accounting for <1% of all cases,^1)^ and digital artery aneurysms are even rarer. In a systematic review of MEDLINE and Embase, Sheikh et al. identified 21 true aneurysms, 26 pseudoaneurysms, and 2 mycotic aneurysms.^2)^ We subsequently performed a MEDLINE search of reports published thereafter and identified an additional 7 cases. As of 2025, there are 25 reported cases of true aneurysm, 29 pseudoaneurysms, and 2 mycotic aneurysms. These cases were incorporated into an updated comparative summary (**Table 1**).^2–10)^ True aneurysms most often presented with compressive symptoms, while pseudoaneurysms were more frequently associated with ischemia and embolic complications. As shown in **Table 1**,^2–10)^ the presenting symptoms of true aneurysms included severe pain (2/25, 8.0%), tenderness (11/25, 44.0%), and hypoesthesia (4/25, 16.0%), whereas pseudoaneurysms most often presented with severe pain (9/29, 31.0%), tenderness (11/29, 37.9%), and hypoesthesia (5/29, 17.2%), suggesting a stronger association with ischemic or embolic events. In the present case, the patient’s chief complaints of mild tenderness and a pulsatile mass were more indicative of a true aneurysm. True aneurysms are most often attributed to chronic mechanical irritation or blunt trauma, often of occupational origin (e.g., metalwork, radiography, and golfing). Other causes include congenital anomalies, inflammatory disorders, atherosclerosis, and idiopathic factors.^2,3,8)^ Digital artery aneurysms may mimic epidermoid cysts, arteriovenous fistulas, foreign-body granulomas, ganglion cysts, or schwannomas, and intraoperative diagnosis is not unusual.^9)^ Bouvet et al. proposed a diagnostic algorithm for hand aneurysms and recommended ultrasonography for palpable lesions, angiography for acute ischemia, and computed tomography or magnetic resonance angiography in the absence of ischemic signs.^10)^ No cases of rupture have been reported, and no consensus on treatment indications has been established. However, in digital artery aneurysms, it is often difficult to distinguish between true and pseudoaneurysms preoperatively. Because pseudoaneurysms carry a higher risk of ischemia, excision is frequently chosen for both symptom relief (pain, numbness, pulsatile mass, cold sensation, cyanosis) and diagnostic confirmation. Treatment options include ligation and excision alone, excision with primary repair, or reconstruction using vein or arterial grafts. When the collateral circulation is sufficient, simple excision with ligation is generally adequate; however, preoperative imaging or digital Allen’s test should be conducted to confirm collateral development.^2,3,8)^ When the collateral circulation is insufficient, treatment options include conservative management, simple ligation and excision with the risk of ischemic complications or even amputation, or technically demanding microvascular reconstruction. Although conservative management has rarely been reported, surgical treatment is generally considered the realistic option. In such cases, thorough preparation is necessary, including consultation with a plastic surgeon experienced in microvascular reconstruction. In this case, angiography confirmed the adequacy of the collateral circulation, allowing successful resection with simple ligation. The patient’s symptoms resolved, and no recurrence was observed at 1 year.

**Table 1:** Digital artery aneurysms: Summary of previous literature

Author	Year	Age	Gender	Type	Symptoms	Imaging	Injury	Location	Surgical indications	Repair	Outcome
Berrettoni	1990	67	M	Mycotic	Painful mass, swelling, 1-week history	US	Infective endocarditis	Index finger	Relieve the pain	E + reconstruction with arterial graft	No sequelae
Bouvet^10)^	2018	39	M	Mycotic	Painful mass	MRI	Previous penetrating trauma and an infected collection	Thumb	Relieve the pain	E + PA	No sequelae
Baruch	1977	21	M	False	Painful mass, hard	X-ray	Glass laceration	Thumb	Relieve the pain	E + L	NR
Hentz	1978	19	M	False	Painful mass, pulsatile mass, following partial amputation of the right index finger	No imaging	Digital amputation	Middle finger	Relieve the pain	E + L + complete amputation of digital stump	No sequelae
Suzuki	1980	69	M	False	Motion limitation, hypoesthesia, ischemic skin changes	Angiography	Machinist, penetrating injury	Thumb	Improve motion limitation	E + L	No sequelae
Sanchez	1982	26	M	False	Tender, pulsatile mass	Angiography	Penetrating injury	Ring finger	Relieve the tenderness	E + PA	No sequelae
Hall	1986	24	M	False	Painful mass, throbbing, 5 days post-injury	Angiography	Penetrating injury	Little finger	Relieve the pain	Repair	No sequelae
Gracia	1987	70	M	False	Numbness, pulsatile mass, 3 weeks following a knife injury	NR	Penetrating injury	Middle finger	Improve numbness	E + L	No sequelae
Ho	1987	NR	NR	False	NR	NR	Puncture wound	Thumb	NR	E + L	No sequelae
Ho	1987	NR	NR	False	NR	NR	Penetrating injury	Little finger	NR	E + L	No sequelae
Tyler	1988	57	F	False	Painful, pulsatile mass, median nerve compression, intermittent cyanosis	Angiography	Opened tins by banging the palm of her hand on the opener for several years	First CPDA	Relieve the pain	E + vein graft	No sequelae
Brunelli	1988	27	M	False	Tender, non-pulsatile mass	X-ray	Crush injury	Middle and ring finger	Relieve the tenderness	E + reconstruction with IVG	No sequelae
Montoya	1991	23	M	False	Painful, pulsatile mass, hypoesthesia, cyanotic, 18 days post-injury	No imaging	Manual worker, penetrating injury	Little finger	Relieve the pain	E + L	No sequelae
Shidayama	1992	13	F	False	Tender, pulsatile mass, 1-week post-injury	No imaging	Penetrating injury	Middle finger	Relieve the tenderness	E + L	No sequelae
Bianchi	1993	70	M	False	Non-pulsatile mass, gradually enlarging for 15 years following penetrating trauma	Angiography	Penetrating injury	Middle finger	NR	E + L	NR
Yajima	1995	58	F	False	Painful, pulsatile`mass	No imaging	Cut	CPDA	Relieve the pain	E + PA	No sequelae
Yasuda	1996	NR	M	False	NR	US	Softball catcher	Thumb	NR	NR	NR
Cromheecke	1997	69	M	False	Tender, pulsatile mass	Angiography	Screwdriver injury	Second CPDA	Relieve the tenderness	Conservative	No sequelae
Abouzahr	1997	6	M	False	Tender, pulsatile mass, violaceous, 10 days post-injury	MRA	Penetrating injury	Index finger	Relieve the tenderness	E + L	No sequelae
Simeonov	1998	4	M	False	Enlarging mass, bleeding	No imaging	Penetrating injury	Second CPDA	Rupture	E + L	NR
Khan	1998	70	M	False	Tender mass, swelling	No imaging	Penetrating injury	Middle finger	Relieve the tenderness	E + L	No sequelae
Ballas	2006	40	M	False	Non-pulsatile mass, fixed	MRA	Textile factory worker, hammer injury, partial factor 8 deficiency	Index finger	NR	E + L	No sequelae
Miyamoto	2009	16	M	False	Tender, enlarging mass, hypoesthesia, 1-year history	MRA	Baseball player	Thumb	Relieve the tenderness	E + PA	No sequelae
Lucchina	2011	43	M	False	Painful mass, 6 weeks post-injury	CTA	Scissor injury	First CPDA	Relieve the pain	E + reconstruction with superficial palmar branch of the radial artery	No sequelae
Chaudhry	2011	54	F	False	Tender, pulsatile mass, firm	US	Dog bite	Index finger	Relieve the tenderness	E + L	No sequelae
Plant	2011	65	F	False	Tender, non-pulsatile mass, fixed	US + angiogram	Penetrating injury	Thumb	Relieve the tenderness	E + PA	No sequelae
Taylor	2012	60	M	False	Painful, pulsatile mass, enlarging, reduced sensation in the radial nerve distribution of the thumb	MRA	Percutaneous trigger finger release	Thumb	Relieve the pain	E + L	No sequelae
Sayit	2017	27	M	False	Tender, pulsatile mass, hypoesthesia, skin atrophy, 1-month post-injury	MRI	Penetrating injury	First CPDA	Relieve the tenderness	E + PA	NR
Zhang^4)^	2023	74	F	False	Painful, pulsatile mass, 2 weeks post-injury	X-ray	Sharp incision injury	Index finger	Relieve the pain	E + L	No sequelae
Yamashiro^5)^	2023	28	M	False	Tender, non-pulsatile mass	US + MRI	Blunt trauma	Middle finger	Relieve the tenderness	E + L	No sequelae
Dukan^6)^	2023	36	M	False	Hypoesthesia, non-pulsatile mass	US	Sharp incision injury	Index finger	Improve hypoesthesia	E + L	No sequelae
Layman	1982	38	M	True	Tender mass, hypoesthesia, 2 years following injury	No imaging	Crush injury	Middle finger	Relieve the tenderness	E + L	NR
Dangles	1984	46	M	True	Painful mass	No imaging	US Navy officer, bowler	Thumb	Relieve the pain	E + L	NR
Turner	1984	52	F	True	Tender mass, hypoesthesia	No imaging	Canteen assistant	Ring finger	Relieve the tenderness	E + L	Complete pain relief, residual hypoesthesia
Ho	1987	NR	NR	True	NR	NR	Unknown	Index finger	NR	E + L	No sequelae
Ho	1987	NR	NR	True	NR	NR	Unknown	Little finger	NR	E + L	No sequelae
Ho	1987	NR	NR	True	NR	NR	Unknown	Index finger	NR	E + L	No sequelae
Ho	1987	NR	NR	True	NR	NR	Volleyball player	Ring finger	NR	E + PA	No sequelae
Trabulsy	1992	21	F	True	Painful, non-pulsatile mass, loss of sensation, reduced 2-point discrimination	No imaging	Telephone operator	Index finger	Relieve the pain	E + L	No sequelae + regained 2-point discrimination
Lanzetta	1992	28	F	True	Tender, pulsatile mass, digit 3 degrees cooler than the opposite hand	Angiography	Volleyball player	Middle finger (x3) + superficial palmar arch	Relieve the tenderness	Conservative	No sequelae
Itoh	1992	8 month	M	True	Pulsatile mass, 1-month history of enlarging	US	Congenital	Third CPDA	NR	E + L	No sequelae
Adant	1994	55	M	True	Severe pain and numbness when trying to grasp objects, present for 1.5 years	No imaging	Metal worker, hemophilia	Thumb	Relieve the pain	E + L	No sequelae
Yajima	1995	69	F	True	Hypoesthesia, non-pulsatile mass	No imaging	Farming	CPDA	Improve hypoesthesia	E + L	No sequelae
Yajima	1995	16	M	True	Hypoesthesia, non-pulsatile mass	No imaging	Baseball	Thumb	Improve hypoesthesia	E + L	No sequelae
Yoshii	2000	29	M	True	Tender, non-pulsatile mass, numbness on ulnar side of finger	MRI	Golfer	Ring finger	Relieve the tenderness	E + L	No sequelae
Taniguchi	2002	47	M	True	Tender mass	No imaging	Radiographer	Thumb	Relieve the tenderness	E + L	No sequelae
Strauch	2004	32	F	True	Tender, pulsatile mass, blue swelling	Angiography	No cause identified	Little finger	Relieve the tenderness	E + reconstruction with IVG	No sequelae
Tanaka	2005	2	F	True	Pulsatile mass, swelling	Angiography	Congenital	Middle finger	NR	E + reconstruction with IVG	No sequelae
Lee^9)^	2006	44	F	True	Tender, non-pulsatile mass	No imaging	Poor fitting wedding ring	Ring finger	Relieve the tenderness	E + L	No sequelae
Quintella	2019	60	M	True	Tender, pulsatile mass	MRA	No cause identified	Middle finger	Relieve the tenderness	E + L	No sequelae
Dean	2019	13 month	M	True	Enlarging, pulsatile mass	Angiography	Congenital	Second CPDA	NR	E + L	No sequelae
Vinnicombe^8)^	2019	44	M	True	Swelling	MRA	Musician, golfer	Second CPDA	NR	E + L	No sequelae
Sheikh^2)^	2020	64	M	True	Pulsatile mass	US + angiography	Electrician	Fourth CPDA	Prevention of thrombosis or rupture	E + PA	No sequelae
Likhitha^3)^	2023	22	F	True	Tender, pulsatile mass	MRA	No cause identified	Little finger	Relieve the tenderness	E + L	No sequelae
Gunawardena^7)^	2025	77	F	True	Tender, pulsatile mass	US + CTA	No cause identified	First CPDA	Relieve the tenderness	E + PA	No sequelae
Nakabori	2025	29	M	True	Tender, pulsatile mass	US + angiography	No cause identified	Index finger	Relieve the tenderness	E + L	No sequelae

NR: not recorded; US: ultrasound scan; MRI: magnetic resonance imaging; MRA: magnetic resonance angiography; CTA: computed tomography angiography; CPDA: common palmar digital artery; E: excision; L: ligation; PA: primary anastomosis; IVG: interposition vein graft; M: male; F: female

## Conclusion

We encountered a rare case of a true digital artery aneurysm. In cases where the collateral circulation is sufficient, simple ligation and excision are safe and effective.
